# 3D Printing Hydrogel-Based Soft and Biohybrid Actuators: A Mini-Review on Fabrication Techniques, Applications, and Challenges

**DOI:** 10.3389/frobt.2021.673533

**Published:** 2021-04-29

**Authors:** Wenhuan Sun, Saul Schaffer, Kevin Dai, Lining Yao, Adam Feinberg, Victoria Webster-Wood

**Affiliations:** ^1^ Biohybrid and Organic Robotics Group, Department of Mechancial Engineering, Carnegie Mellon University, Pittsburgh, PA, United States; ^2^ Morphing Matter Lab, Human-Computer Interaction Institute, School of Computer Science, Carnegie Mellon University, Pittsburgh, PA, United States; ^3^ Regenerative Biomaterials and Therapeutics Group, Department of Materials Science and Engineering, Carnegie Mellon University, Pittsburgh, PA, United States

**Keywords:** 3D printing, hydrogel, biohybrid actuator, fabrication, actuation mechanism

## Abstract

Stimuli-responsive hydrogels are candidate building blocks for soft robotic applications due to many of their unique properties, including tunable mechanical properties and biocompatibility. Over the past decade, there has been significant progress in developing soft and biohybrid actuators using naturally occurring and synthetic hydrogels to address the increasing demands for machines capable of interacting with fragile biological systems. Recent advancements in three-dimensional (3D) printing technology, either as a standalone manufacturing process or integrated with traditional fabrication techniques, have enabled the development of hydrogel-based actuators with on-demand geometry and actuation modalities. This mini-review surveys existing research efforts to inspire the development of novel fabrication techniques using hydrogel building blocks and identify potential future directions. In this article, existing 3D fabrication techniques for hydrogel actuators are first examined. Next, existing actuation mechanisms, including pneumatic, hydraulic, ionic, dehydration-rehydration, and cell-powered actuation, are reviewed with their benefits and limitations discussed. Subsequently, the applications of hydrogel-based actuators, including compliant handling of fragile items, micro-swimmers, wearable devices, and origami structures, are described. Finally, challenges in fabricating functional actuators using existing techniques are discussed.

## 1 Introduction

Recent advances in 3D printing have enabled the production of customizable hydrogel-based actuators with a variety of applications. Hydrogels are hydrophilic and porous crosslinked polymer networks whose mechanical, chemical, and stimulation-responsive properties can be tuned based on composition and manufacturing processes (Shi et al., 2019). Such properties enable applications of hydrogels in several domains, including tissue engineering (Billiet et al., 2012), drug delivery (Li and Mooney, 2016), wound dressings (Varaprasad et al., 2020), and soft robotics (Lee et al., 2020; Wallin et al., 2018). Recent reviews are available on the use of hydrogels in biomedical applications (Banerjee et al., 2018; Champeau et al., 2020), polymeric shape memory hydrogels and hydrogel actuators (Shang et al., 2019), and biomimetic hydrogel actuators (Le et al., 2019). Readers interested in comprehensive reviews on hydrogel-based 3D printing for biomedical applications can refer to (Li et al., 2020). However, a focused synopsis of the fabrication techniques and challenges for 3D printing hydrogel-based actuators has not been previously reported.

To address this gap, this mini-review presents recent advancements in 3D printing for fabricating hydrogel actuators, either as a standalone manufacturing process or integrated with traditional fabrication techniques, where actuators are defined as any components that perform defined movements or geometric changes. Here, we present characteristic examples of existing 3D printing techniques for hydrogel actuator fabrication based on their roles in the fabrication process: direct 3D printing; mixed-mode 3D printing, where 3D printed hydrogel structures undergo additional post-printing steps; and the use of 3D printing to fabricate intermediate tools. Additionally, we discuss the strengths and limitations of 3D printed hydrogel actuators based on their actuation modalities. Applications of 3D printed hydrogel actuators are subsequently surveyed. Through this discussion, we provide a focused survey on the challenges and limitations in fabricating hydrogel actuators using existing 3D printing techniques to inspire future hydrogel fabrication advances that improve the performance of hydrogel-based actuators.

## 2 3D Printing Techniques for Hydrogel Actuator Fabrication

3D printing enables hydrogel actuator fabrication either through direct printing of actuator structures, mixed-mode printing of hydrogels with other functional materials, or through printing intermediate tools. Several direct 3D printing technologies have emerged including inkjet-based, laser-based, and extrusion-based approaches. Other fabrication techniques can be added to direct printing to create mixed-mode fabrication platforms, such as combining 3D printing and hydrogel electrospinning. Additionally, 3D printing can be used to rapid prototype molds for casting hydrogels. Each approach has challenges and advantages that should be considered when selecting fabrication techniques.

### 2.1 Direct 3D Printing of Hydrogel Actuators

#### 2.1.1 Inkjet-Based 3D Printing

**FIGURE 1:**
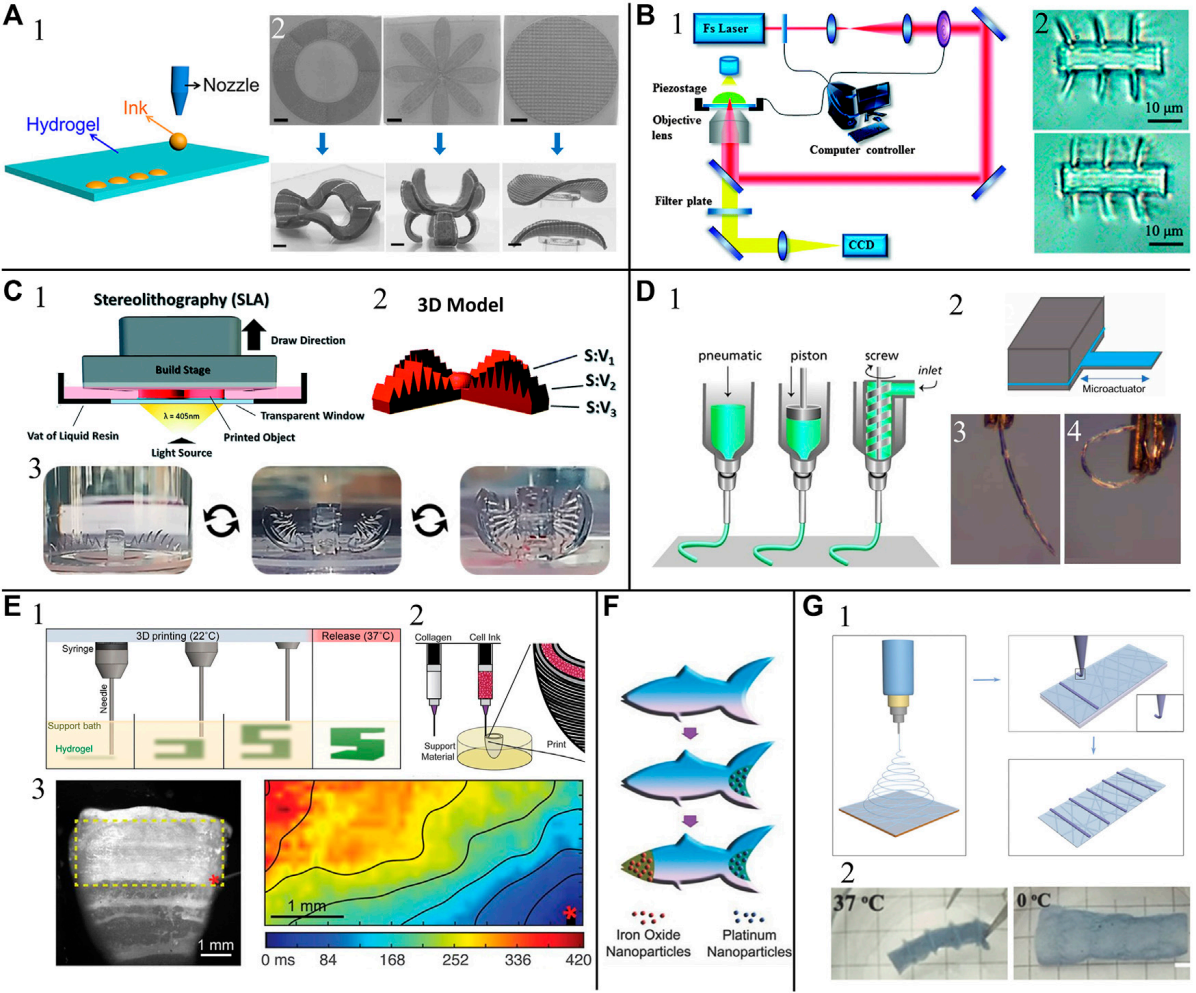
Examples of hydrogel actuators fabricated with **(A)** inkjet printing, **(B)** two-photon polymerization printing (2PP), **(C)** stereolithography (SLA), **(D)** extrusion-based printing, **(E)** embedded printing, and **(F,G)** mixed-mode 3D printing. **(A1)** The inkjet printing process. **(A2)** Hydrogel actuators with inkjet printed patterns to achieve controlled 3D deformation. Adapted from (Peng and Wang 2018) ©2018 John Wiley and Sons, Inc. **(B1)** A typical 2PP process induced by a near-infrared femtosecond laser. Reprinted from (Xing et al., 2015) ©2015 the Royal Society of Chemistry. **(B2)** 2PP printed hydrogel actuator in water **(top)** and in 1 M NaCl solution **(bottom)**. Reprinted from (Xiong et al., 2011) ©2011 the Royal Society of Chemistry **(C1)** The SLA process **(C2)** 3D model of an SLA printed hydrogel actuator with varying surface area to volume ratio. **(C3)** Osmotically driven actuation of the actuator. Adapted from (Odent et al., 2019) ©2019 the Royal Society of Chemistry. **(D1)** Common extrusion-based printing process. Reprinted from (Malda et al., 2013) ©2013 John Wiley and Sons, Inc. **(D2)** A bending actuator with 3D printed humidity-responsive hydrogel layer (blue) with water vapor induced actuation **(D3-4)**. Adapted from (Tyagi et al., 2020) ©2020 The Authors under the CC-BY 4.0 license. **(E1)** Embedded printing process where the thermo-responsive support bath exhibits Bingham plastic properties during printing and melt at raised temperature for printed component release. Reprinted from (Hinton et al., 2015) ©2015 The Authors under the CC-BY 4.0 license **(E2)** Fabrication process of an embedded printed human cardiac ventricle model using collagen and cell ink. **(E3)** **(left)** Point stimulation of the model stained with calcium-sensitive dye. **(right)** Color-coded calcium mapping of a subregion showing anisotropic calcium wave propagation during stimulated contraction. Adapted from (Lee et al., 2019) ©2019 AAAS. **(F)** Fabrication of a 3D printed microfish with encapsulated magnetic nanoparticles for motion control and catalytic Pt nanoparticles for self-propulsion powered by H_2_O_2_ decomposition. Adapted from (Zhu et al., 2015) ©2015 John Wiley and Sons, Inc. **(G1)** A hydrogel actuator fabricated with hydrogel electrospinning and 3D printing. **(G2)** The actuator exhibits temperature-dependent curvature. Adapted from (Chen et al., 2018) ©2018 John Wiley and Sons, Inc. All figures are used with permission.

Inkjet-based hydrogel 3D printing uses computer-controlled layer-by-layer deposition of droplets onto a substrate (Figure 1A1) (Axpe and Oyen, 2016). A variety of shape changing hydrogel actuators that respond to external stimuli have been inkjet printed (Peng and Wang, 2018). For example, utilizing the swelling response of inkjet printed UV-curable polyacrylated monomers that undergo layer-by-layer UV-polymerization, water-swellable joints can be fabricated to enable modular 4D self-reconfiguration following printing (Raviv et al,. 2014). Alternatively, inkjet printers can be used to introduce reactive inks into hydrogel systems to induce locally active regions. For example, metal ion ink to induce anisotropic crosslinking density and controlled deformation during swelling (Figure 1A2) (Peng et al., 2017), and reactive catalyst ink made of bis(2,2′-bipyridine)-4′-methyl-4-carboxybipyridine-ruthenium N-succinimidyl ester-bis(hexafluorophosphate) (Ru (sbpy)) to create user-defined reactive regions that experience oxidation state-dependent swelling ratio (Kramb et al., 2014).

Hydrogels with low viscosity of <10 mPa.s (Axpe and Oyen, 2016) are typically used in inkjet printing and the resulting hydrogel actuators have low structural integrity (Zolfagharian et al., 2017a). The aforementioned techniques produced relatively thin and simple hydrogel structures. Fabricating thick hydrogel actuators using inkjet printing remains a challenge. To overcome this limitation, reinforcing material, such as collagen sheets or agarose rods, can be printed as support materials before depositing hydrogel layers (Jakab et al., 2010). Recent advancement in freeform liquid support-based inkjet printing techniques may further lift the geometric constraints on 3D inkjet printed hydrogel actuators. For example, (Christensen et al., 2015) fabricated vascular-like structures with overhanging features by directly inkjet printing hydrogel into a calcium chloride solution, which serves as both a crosslinker and support.

#### 2.1.2 Laser-Based 3D Printing

Hydrogel actuators with 2D geometry can be fabricated with classic photolithography that uses photomasks for geometry control, such as stop-flow lithography (Rehor et al., 2020). Hydrogel structures with complex 3D geometry can be made via laser-based 3D printing techniques from three major categories: laser-induced forward transfer, which uses laser energy to discharge hydrogel droplets from a donor layer onto a substrate (Guillemot et al., 2011); two photon-polymerization (2PP) (Figure 1B1) which initiates hydrogel polymerization through irradiation with near-infrared laser pulses (Billiet et al., 2012); and stereolithography (SLA) (Figure 1C1), which selectively crosslink photo-sensitive monomer resin using scanning UV laser beams (Cvetkovic et al., 2014). A variation of SLA is digital light processing (DLP), which projects the entire image of a layer on the resin using UV (or visible) light from a digital projector (Soman et al., 2013). High resolution SLA provides a variety of tunable fabrication parameters and can be used to fabricate hydrogel actuators with controllable motion through asymmetry. Specifically, anisotropic swelling behavior has been encoded by asymmetric high surface area patterning, asymmetric crosslinking density, and asymmetric chemical composition by tuning SLA parameters (Odent et al., 2019), (Figures 1C2,3). Compared with other hydrogel 3D printing techniques, 2PP shows exceptional spatial resolution and is advantageous for building miniature actuators and robots. For example, (Xiong et al., 2011) fabricated ion-responsive hydrogel microcantilever actuators with swift motion (millisecond response time) and small size (10 μm cantilever length) using 2PP (Figure 1B2).

Laser-based hydrogel 3D printing techniques show great spatial resolution (up to submicron resolution (Ovsianikov et al., 2010; Xing et al., 2015). However, they are typically subject to high costs due to expensive hardware and software and the limitation of printing small features.

#### 2.1.3 Extrusion-Based 3D Printing

Extrusion-based 3D printing dispenses hydrogel filament through a nozzle onto a substrate using a piston, pneumatic pump or screw (Figure 1D1) (Li et al., 2020). With a large selection of open-source and low-cost fabrication hardware and software, extrusion-based 3D printing is considered attractive for hydrogel processing (Li et al., 2020), and many hydrogel actuators fabricated with extrusion printing have been reported. Using a pneumatically driven fluid dispenser, (Tyagi et al., 2020) fabricated a microactuator where an active hydrogel layer that swells upon hydration was bonded with a passive gel layer to create a cantilever structure with humidity-induced bending capability (Figures 1D2–4). With continuous hydrogel filament feeding, extrusion-based 3D printing provides a convenient way to create anisotropic hydrogel features that facilitate directional actuation. For example, (Gladman et al., 2016) printed composite hydrogel actuators with anisotropic swelling behavior by aligning cellulose fibrils along predefined printing pathways. (Cheng et al., 2019) demonstrated the versatility of extrusion-based 3D printing with modified hydrogels by fabricating fluidic and stimulus-activated actuators, including an artificial tendril with phototropic motion.

Extrusion-based 3D printing is a cost-effective hydrogel fabrication technique that is compatible with hydrogel ink with viscosities ranging from 30 to 6 ⋅ 10^7^ mPa ⋅ s (Kyle et al., 2017). Large hydrogel constructs can be printed with moderate spatial resolution. With recent advances in support material for embedded 3D printing, the spatial resolution of extrusion based 3D printed hydrogel actuators can be further improved (see Section 2.1.4).

#### 2.1.4 Embedded 3D Printing

3D printing overhanging features without supporting structures often leads to low print fidelity due to unwanted deformation. This problem is more pronounced for hydrogel actuator fabrication because many of the inks are extremely soft, e.g., alginate and collagen, and can not even support their own weight. Recently, several embedded 3D printing techniques have been proposed to resolve the aforementioned issue by printing modified inks into a support bath (Zhang et al., 2021; Mirdamadi et al., 2020). Notably, (Hinton et al., 2015) printed 3D structures by embedding printed hydrogels within a gelatin-based thermoreversible bi-phase support material, which provides support during printing and liquefies when the temperature is raised post-printing for part retrieval (Figure 1E1), coined Freeform Reversible Embedding of Suspended Hydrogels (FRESH). Using FRESH 2.0 with improved spatial resolution (20 μm filament resolution), (Lee et al., 2019) printed cardiac ventricles with synchronized contractions using human cardiomyocytes and collagen (Figures 1E2,3).

### 2.2 Mixed-Mode 3D Printing and 3D Printing of Intermediate Tools

Combining 3D printing with other fabrication techniques (mixed-mode), allows 3D printed hydrogel actuator fabrication with additional functionality. For example, (Zhu et al., 2015) demonstrated a chemically powered and magnetically guided microfish, where the hydrogel body was fabricated via UV photolithography. Catalytic Pt nanoparticles were then encapsulated at the tail via crosslinking Pt-doped resin to induce self-propulsion via decomposition of H_2_O_2_. Fe_3_O_4_ nanoparticles were loaded into the head with a similar method for magnetic control (Figure 1F). In another example of mixed-mode fabrication, (Chen et al., 2018) combined hydrogel electrospinning and extrusion-based hydrogel printing to fabricate actuators with temperature-responsive bending behavior induced by the difference in properties of electrospun and 3D printed hydrogels (Figure 1G). (Cvetkovic et al., 2014) demonstrated a biobot whose hydrogel body was SLA 3D printed and actuated by a strip of engineered mammalian skeletal muscle cast over the printed hydrogel body.

In addition to direct 3D printing, hydrogel actuators can be made with methods that indirectly involve 3D printing. For example, (Yuk et al., 2017) fabricated hydraulic hydrogel actuators whose bodies were made by casting within 3D printed molds. In addition to mold fabrication for hydrogel casting, components of hydrogel actuators have been 3D printed using non-hydrogel materials with desired properties. For example, (Mestre et al., 2019) fabricated a biohybrid actuator using a 3D printed PDMS skeleton that serves as an actuation force indicator and structural support. Combining 3D printing with other fabrication techniques facilitates rapid prototyping of actuators (Yuk et al., 2017) and integration of different materials that enable new actuation modalities (Zhu et al., 2015). However, added fabrication complexity can incur challenges, such as the necessity to switch between resins with different additives (Zhu et al., 2015) and the need to bond cast components (Yuk et al., 2017).

## 3 Actuation Modalities

Hydrogel actuators commonly rely on heterogeneous structure and/or stimuli to generate meaningful movement (Zhang and Khademhosseini, 2017). Heterogeneous structure may be generated from rational design of hydrogel geometry or from material property gradients created by dopants within the hydrogel (Odent et al., 2019). Heterogeneous stimulation may come from external humidity or pH gradients or optical, thermal, or electrical stimuli (Zolfagharian et al., 2017a). Actuation of these hydrogels occurs due to thermodynamic imbalance between osmosis and hydrophobicity of hydrogel polymers which causes swelling and de-swelling (Peng and Wang, 2018). However, other actuation modalities such as cell-powered, pneumatic/hydraulic, acoustic, and magnetic actuation do not typically rely on swelling behavior. Readers interested in a further review of hydrogel-based actuation mechanisms, including actuators that have not been 3D printed or fabricated with 3D printed tooling, may also be interested in (Erol et al., 2019).

### 3.1 Osmotic Actuation (pH, Humidity, Ionic, Thermal, Optical)

Many of the actuation methods of stimuli-responsive hydrogels rely on structural swelling and de-swelling driven by osmotic-pressure changes in response to external stimuli such as pH (Nadgorny et al., 2016), humidity, temperature, electric field, and optical lasers (Yuk et al., 2017). These actuation methods are often used in 4D printing, where hydrogel structures deform over time from their original geometry into new configurations with or without further active actuation (Adam et al., 2021; Momeni et al., 2017; Spiegel et al., 2020). To mitigate the heavy computational costs of simulating 4D printed configurations, (Huang et al., 2020) proposed a modular design of shape-morphing structures with discretized building blocks using a pH-responsive bilayer hydrogel (Jin et al., 2020). For actuating hydrogels using electric field or electrolytes, hydrogel polymers can be created with ionic dopants or precursors (Zolfagharian et al., 2017b). Alternatively, hydrogels can also be actuated without external electric field by changing their chemical environment and building multi-layer hydrogels where each layer has a different swelling response to an electrolyte or chemical solution (Zheng et al., 2018).

Hydration and dehydration cycles can drive structural expansion and contraction of hydrogel actuators due to water absorption from external humidity or immersion in water. This actuation modality is often used to create bilayer and trilayer bending hydrogel actuators. For example, (Rivera et al., 2020) fabricated a hydrogel-textile bilayer composite by 3D printing κ-carrageenan, a nontoxic hydrogel derived from red seaweed that swells in response to water, onto textile substrates. By tuning hydrogel patterns and concentrations, smart textile actuators with programmed actuation states were achieved. (Wang et al., 2017) 3D printed a *B. subtilis* cell-agar hydrogel mixture that experienced volume change during hydration and dehydration by utilizing cells’ hygromorphic phenomenon, where a cell’s size can change by 50% when varying the relative humidity. Similarly, (Yao et al., 2015) printed natto cell-water solution onto humidity-inert textile substrates to create smart wearables that open up ventilation holes in response to raised skin temperature. In addition, (Lv et al., 2018) used 2PP to create hydrogels with microstructure arrays of various geometries to mimic stomata found in plants. Hygroscopic swelling and deswelling causes the stomata microstructures to close and open.

Temperature changes and temperature gradients can be used to actuate hydrogels with heterogeneous lower or upper critical solution temperature (LCST, UCST) (Hua et al., 2019) and hydrogels with temperature-dependent moisture content (Hamedi et al., 2016). (Odent et al., 2019) used SLA to 3D print multi-layer hydrogel actuators that opened and closed between 25–50°C, as well as another hydrogel actuator that was controlled by changes in pH. (Lin et al., 2020) 3D printed a bilayer hydrogel actuator with α-cyclodextrin polyrotaxane that both deflected and changed opacity when subjected to temperature changes.

Optical lasers can also be used to control hydrogel actuators, commonly with a photothermal process using photoresponsive hydrogels and near-infrared light (Watanabe et al., 2002; Hippler et al., 2019). With embedded light-absorbing particles, such as graphene oxide nanoparticles (Zhao et al., 2018; Breuer et al., 2019), copper sulfide nanoparticles (Pan et al., 2021), and gold nanorods (Nishiguchi et al., 2020), the photothermal process converts light energy to heat and follows the same LCST or UCST process as thermally responsive hydrogel actuators, but may be more precisely controlled with optical stimuli (Zheng et al., 2020). The swelling properties of optically stimulated, thermo-responsive hydrogels can be tuned by varying printing density to achieve ultrafast actuation (Nishiguchi et al., 2020).

### 3.2 Cell-Powered Actuation

Hydrogels are among the most common scaffold material in tissue engineering (Mantha et al., 2019). As a consequence, with the growth of biohybrid robotics research over the past decades (Webster-Wood et al., 2017), numerous cell-powered, hydrogel actuators have been developed. Biohybrid devices rely on contraction of engineered or explanted tissue to drive actuation (Ricotti et al., 2017; Webster-Wood et al., 2017; Sun et al., 2020). Cells can be cultured as monolayers to drive bending-based actuation of bilayer cantilevers (Marzban and Yuan, 2016; Chan et al., 2012; Ricotti and Fujie, 2017), or can be cultured as three dimensional tissues using casting (Cvetkovic et al., 2014; Raman et al., 2016) or 3D printing (Mestre et al., 2019). Although many example of mobile hydrogel devices are now available in the literature (Ricotti et al., 2017; Webster-Wood et al., 2017; Sun et al., 2020; Morimoto and Takeuchi, 2020), challenges remain both in identification of optimal hydrogel composition and stiffness, and in scaling up cell-powered actuators for macro-scale applications (Won et al., 2020). Despite these challenges, cell-powered actuation of hydrogels posses many unique advantages through the inclusion of material that is self-healing, renewable, and adaptable.

### 3.3 Pneumatic/Hydraulic Actuation

Pneumatic and hydraulic actuation is based on pressure gradient across an actuator membrane induced by a gas or fluid. Compared to hydrogel actuators with osmotic swelling/de-swelling-based actuation and cell-powered actuation, hydraulically driven hydrogel actuators are less common but show higher actuation force and/or speed (Yuk et al., 2017). These actuators have direct applications in robotics. For example, (Cheng et al., 2019) fabricated hydraulically driven tentacles using alginate-doped acrylamide hydrogel precursors. More recently, (Mishra et al., 2020) demonstrated SLA 3D-printed hydrogel bending actuators that can be pneumatically and hydraulically actuated while maintaining a stable actuator temperature via autonomic perspiration to achieve a trade-off between actuation efficiency and cooling capacity.

### 3.4 Magnetic Actuation

Hydrogels with embedded ferromagnetic nanoparticles can be actuated remotely using an external magnetic field without need for direct contact (Podstawczyk et al., 2020). (Chin et al., 2017) used a combination of photolithography and casting to demonstrate magnetically actuated hydrogel drug delivery devices and gated valves with iron oxide nanoparticles. Additionally, magnetic particle-embedded hydrogels can be 2PP printed into miniature helices with swimming capabilities under rotational magnetic fields (Ceylan et al., 2019; Bozuyuk et al., 2018; de Marco et al., 2019; Park et al., 2019; Cabanach et al., 2020) fabricated similar structures by casting magnetic nanoparticles-gelatin mixture in 2PP printed molds. Alternatively, such microrobots can be made by surface coating of 2PP printed hydrogel helix with magnetic nanoparticles (Wang et al., 2018; Koepele et al., 2020; Dong et al., 2020).

### 3.5 Acoustic Actuation

Similar to magnetic actuation, acoustic energy can be utilized to remotely manipulate micro/nano objects in fluid with high precision (Matouš et al., 2019; Guo et al., 2016; Ozcelik et al., 2018; Ren et al., 2019; Tao et al., 2019; Fornell et al., 2019). Hydrogel structures that are acoustically stimulated at their resonant frequency can manipulate fluids for mixing (Orbay et al., 2018) or generating fluid flow (Kaynak et al., 2020). In addition, (Son et al., 2020) constructed a remotely controlled hybrid gripper with both magnetic and acoustic actuation, where the acoustically responsive hydrogel converted ultrasound energy to heat for temperature-sensitive swelling/de-swelling.

## 4 Soft Robotic Applications of 3D Printed Hydrogel Actuators

The actuation modalities described above have been used for myriad applications (Li et al., 2020). The most relevant to the field of soft robotics are manipulation and locomotion. Additionally, these actuators have use cases in wearables and as origami structures.

### 4.1 Manipulators and Locomotors

**FIGURE 2:**
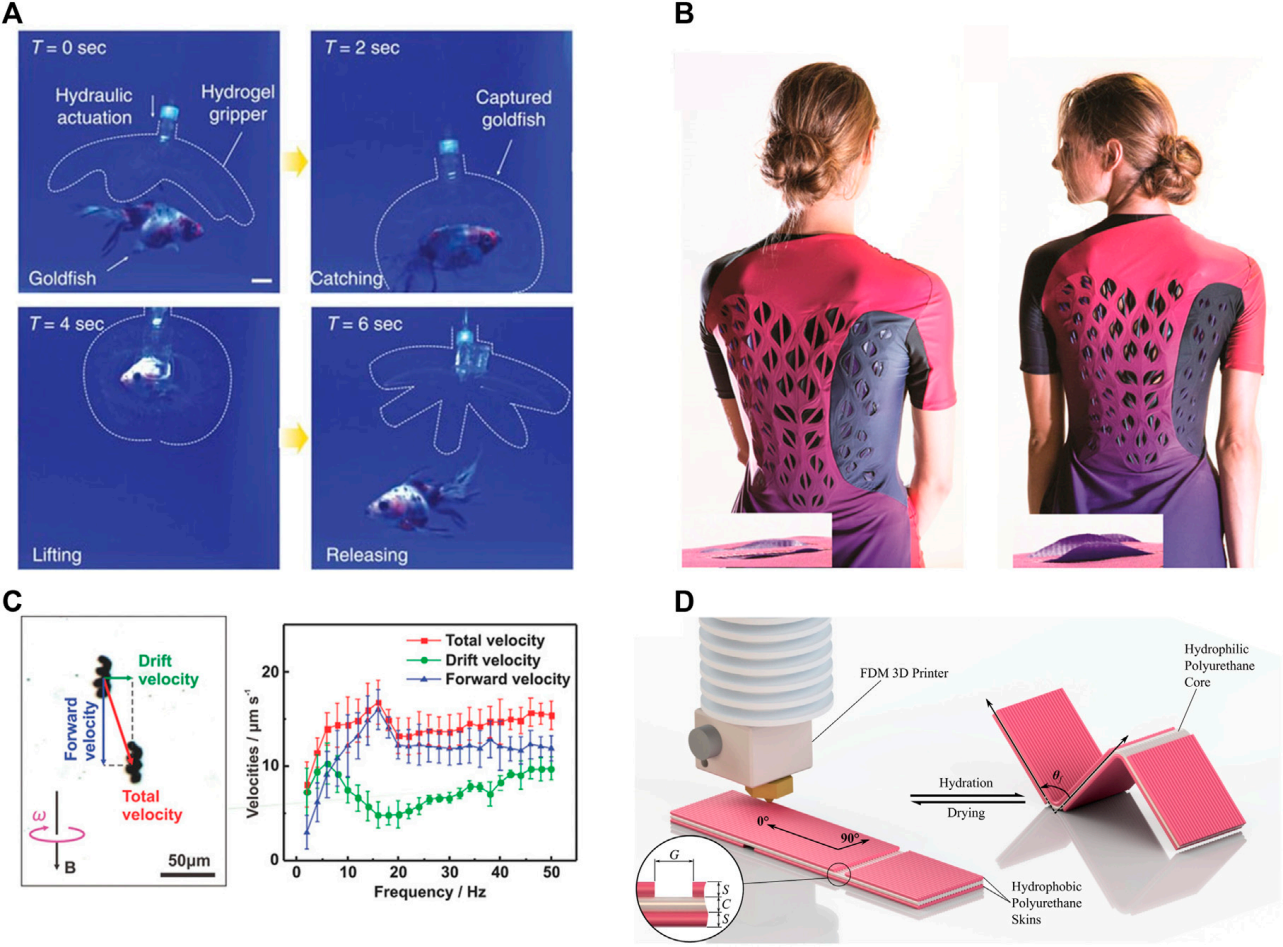
Examples of various applications of hydrogel actuators whose fabrication involves 3D printing. **(A)** A hydrogel gripper catches, lifts and releases a live goldfish without harm. Dotted lines indicates the boundaries of transparent hydrogel structure. Adapted from (Yuk et al., 2017) ©2017 The Authors under the CC-BY 4.0 license. **(B)** Garment prototype with sweat activated cooling ventilation showing flat ventilation flaps before exercise **(left)** and curved ventilation flaps after exercise **(right)**. Adapted from (Wang et al., 2017) ©2017 The Authors under the CC-BY 4.0 license. **(C)** A hydrogel helical micro swimmer actuated by magnetic field **(left)**. Swimming velocities of the microswimmers at different rotational frequency at an applied magnetic rotating field of 8 mT. Adapted from (Wang et al., 2018) ©2018 John Wiley and Sons, Inc. **(D)** 3D printed tri-layer actuator as modularized origami structure consist of hydrophobic polyurethane top and bottom skins (pink), with a hydrophilic polyurethane core (white) **(left)**. It shows hydration-dependent bending at skin gaps **(right)**. Adapted from (Baker et al., 2019) ©2019 The Authors under the CC-BY 4.0 license. All figures are used with permission.

Devising a gripper to demonstrate the functionality of a fabrication technique has become a mainstay in the field of soft robotics, and the subfield of 3D printed hydrogel actuators is no exception. The main mode of operation for these finger-like grippers is the reversible bending of compliant beams to grasp and release objects. For example, (Mishra et al., 2020) made use of a variably porous hydrogel layer that enabled sweating of their finger actuator when introduced to hot environments to facilitate thermoregulation, which is vital for both biological and engineered systems to function at peak power for prolonged durations. A morphologically similar gripper was described by (Yuk et al., 2017) and was able to grasp and move a live fish using its fingers while remaining nearly transparent (Figure 2A), made possible by the low stiffness and visibility of the hydrogels that comprised the gripper.

3D printed hydrogel actuators have also been used to power locomotion in soft robots. Similar to grippers, these locomotory actuators operate by the cyclic deformation of beams, as demonstrated by (Han et al., 2018) for walking and (Tognato et al., 2019) for swimming. In the case of cell-powered systems, the 3D printed hydrogel serves as a compliant structure that is bent by living materials to achieve locomotion, as demonstrated by (Pagan-Diaz et al., 2018; Mestre et al., 2019; Cvetkovic et al., 2014). Beyond walking locomotion, (Wang et al., 2018) demonstrated a micro-swimming robot using 3D printed hydrogel actuators (Figure 2C).

### 4.2 Wearables and Origami Devices

Skin-interfacing materials require low stiffness, making 3D printed hydrogel actuators well-suited as wearable devices. These devices provide users with functional and esthetic utility, as shown by (Wang et al., 2017) where the sweat of the wearer triggers the opening of cooling vents on clothing (Figure 2B). Similarly, (Rivera et al., 2020) demonstrated a hydrogel-textile bilayer actuator that contracts when dehydrated, with applications as weather-triggered signage.

Origami structures couple the ease of planar fabrication methods with the geometric complexity achievable through folding. Printed as flat structures, origami-actuated hydrogels can assume complex 3D forms, as demonstrated by (Naficy et al., 2017; Gladman et al., 2016; Baker et al., 2019), from boxes to helices. These compliant, biocompatible, biodegradable, foldable structures morph between distinct configurations in response to external stimulus (Figure 2D).

## 5 Discussion

Many 3D printing techniques are capable of creating hydrogel structures with user-defined geometry and responsiveness to environmental cues. Using combinations of 3D printing methods and hydrogels, functioning actuators with a variety of actuation modalities and potential applications have been fabricated. This mini-review surveyed the applications of 3D printing for hydrogel actuator fabrication and discussed their corresponding strengths and limitations. However, the applications of these actuators in soft robotics beyond the lab remain limited due, in part, to the mechanical properties of hydrogels and the available spatial resolutions of specific printing techniques. To address these challenges and broaden the areas of application for 3D printing hydrogel actuators, future research is needed to integrate multiple 3D fabrication techniques and utilize the combined strengths of individual methods in a single platform. For example, a hydrogel robot may require features with different spatial resolutions at different locations on the robot body. To accomplish this, an extrusion-based method could print bulk material for features with low resolution and laser-based methods could make features with high resolution. Such integrated platforms could also facilitate multi-material integration for additional functionality. For example, embedded printing methods could introduce traces of conductive and stimuli-responsive material into hydrogel systems to create actuators with embedded sensors. Addressing these challenges through innovative fabrication techniques will further improve the performance of hydrogel-based actuators for applications in soft robotics.
